# Successful Primary Closure of Cricotracheostomy Fistula after Cardiac Surgery: Usefulness of Cricotracheostomy in Post-Sternotomy

**DOI:** 10.70352/scrj.cr.25-0524

**Published:** 2026-01-09

**Authors:** Mitsunobu Toyosaki, Toshiharu Nakama, Mamoru Orii, Ako Takusagawa, Hiroki Moriuchi, Kouhei Narayama, Akihiko Yamauchi, Masahiro Tamashiro, Junichi Sasaki

**Affiliations:** 1 Department of Critical Care Medicine, Yuuai Medical Center, Tomigusuku, Okinawa, Japan; 2 Department of Emergency and Critical Care Medicine, Keio University School of Medicine, Tokyo, Japan; 3 Department of Cardiovascular Surgery, Yuuai Medical Center, Tomigusuku, Okinawa, Japan

**Keywords:** tracheostomy, sternal wound infection, cardiac surgery

## Abstract

**INTRODUCTION:**

Patients with complications requiring prolonged mechanical ventilation after cardiac surgery may need a tracheostomy. However, a high rate of sternal wound infection (SWI) after tracheostomy is concerning. Cricotracheostomy is a novel method used to achieve a higher tracheal incision than that using conventional surgical tracheostomy and is often performed by otolaryngologists in patients with anatomical abnormalities. However, it may affect speech and is generally recommended only in cases where tracheal stoma closure is not considered. In addition, its usefulness after cardiac surgery has not been fully verified.

**CASE PRESENTATION:**

A female patient in her 60s was admitted for acute aortic dissection with cardiac tamponade and underwent ascending aortic replacement and pulmonary artery patch formation. On POD 7, the patient was extubated. Pericardial fenestration was performed because of pericardial effusion. On POD 14, the patient was re-intubated owing to inability to expel sputum. On POD 16, a tracheostomy was performed. A cricotracheostomy was performed to avoid SWI and because of her anatomical abnormality—a low-lying larynx. No major complications, including SWI, were observed after cricotracheostomy. On POD 41, the patient was completely weaned off the ventilator. Primary closure of the cricotracheostomy fistula was performed on POD 47, and the patient had no problems with speech or swallowing.

**CONCLUSIONS:**

This case highlights the usefulness of cricotracheostomy after cardiac surgery. Cricotracheostomy may be an optimal method for preventing SWI and preserving vocal function after cardiac surgery.

## Abbreviations

AAD
: acute aortic dissection
PDT
: percutaneous dilatational tracheostomy
ST
: surgical tracheostomy
SWI
: sternal wound infection

## INTRODUCTION

Some patients with complications due to infection, cardiac failure, respiratory distress, or other complications after cardiac surgery require prolonged mechanical ventilation and tracheostomy.^1)^ Tracheostomy is a safe, comfortable, and commonly used method for prolonged mechanical ventilation. It was first performed in 1964 in a patient who had undergone cardiac surgery.^2)^ Early tracheotomy improves outcomes in patients who require tracheotomy after cardiac surgery.^3)^ However, the rate of SWI is known to increase in patients who undergo tracheotomy following cardiac surgery,^4)^ which is a concern.

**Fig. 1:**
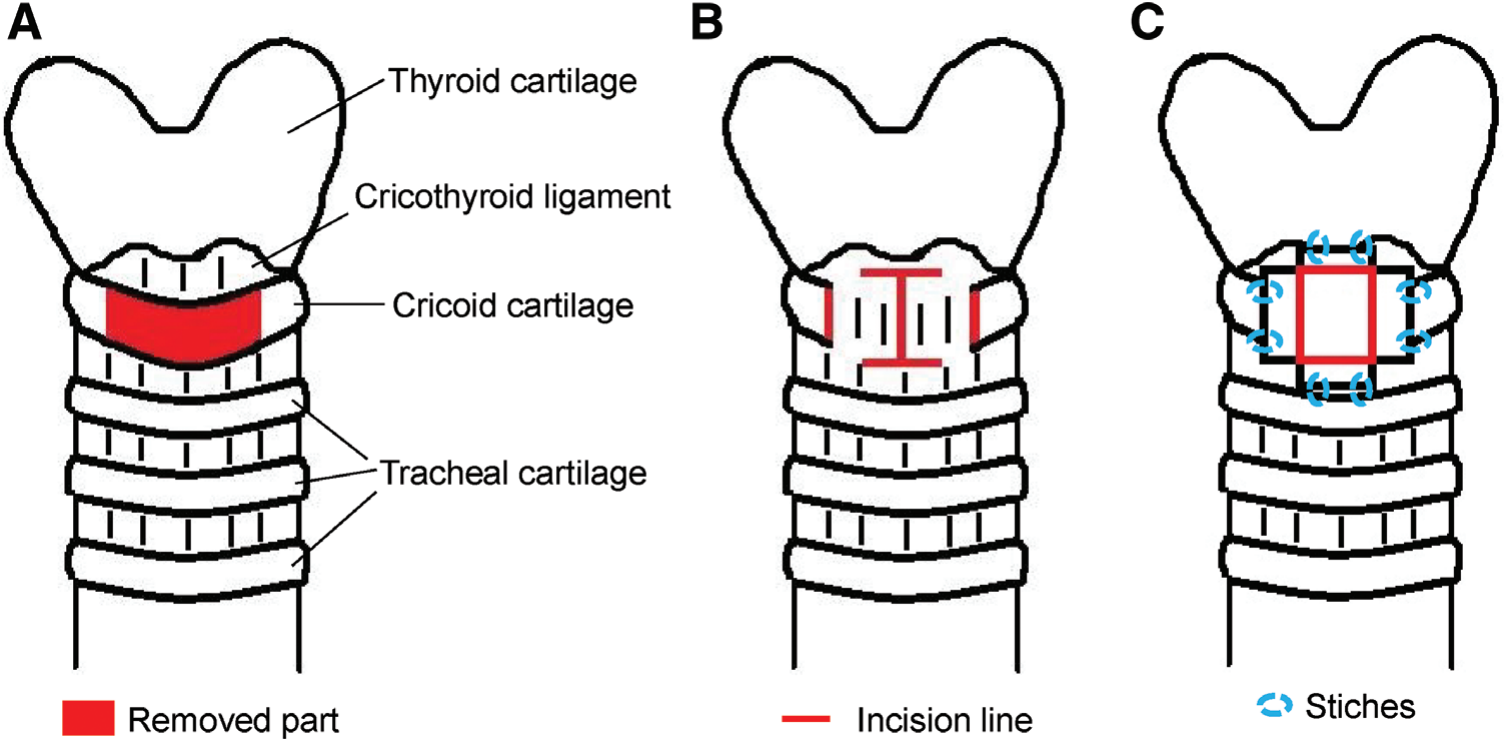
Cricotracheostomy method. (**A**) Front of the cricoid cartilage was removed. (**B**) A horizontal H-shaped incision was made in the ligament. (**C**) Eight stitches were placed between the ligament and the skin around the fistula. Cited and adapted from Toyosaki et al.^6)^

Cricotracheostomy is a surgical procedure in which a part of the cricoid cartilage is removed to achieve a higher incision than that using the conventional method. It is often performed in patients with anatomical abnormalities^5)^ (**Fig. 1** ^6)^). Cricotracheostomy is often performed by otolaryngologists, and its usefulness in patients with anatomical abnormalities is becoming evident.^7,8)^ However, the usefulness of cricotracheostomy after cardiac surgery has not been fully investigated. In addition, the effects of this surgical procedure on swallowing and speech function after cardiac surgery need to be examined.

We hypothesized that SWI in patients undergoing tracheostomy after cardiac surgery is due to the proximity of the sternal wound to the conventional tracheostomy wound. Cricotracheostomy makes it possible to place the wound at a distance from the sternal wound, which may prevent SWI. Here, we describe the successful primary closure of a cricotracheostomy fistula in a patient after ascending aortic replacement for AAD and report the surgical outcomes, including swallowing and speech function.

## CASE PRESENTATION

**Fig. 2:**
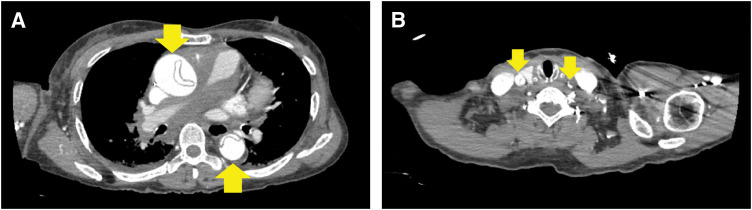
CT with venous enhancement. (**A**) Dissection from the ascending aorta (yellow arrows). (**B**) Dissection of both common carotid arteries; the left was almost completely blocked (yellow arrows).

A woman in her 60s with a diagnosis of AAD with cardiac tamponade was transported via helicopter from an island hospital to our hospital for emergency surgery. The patient complained of difficulty in breathing during dialysis at a clinic and was transported to the island hospital. As the patient was in shock and because cardiac ultrasonography revealed cardiac tamponade, pericardiocentesis was performed immediately. A CT scan revealed Stanford type A AAD. Both common carotid arteries were dissected, and the left carotid artery was almost completely blocked (**Fig. 2**). The patient had undergone dialysis for end-stage renal failure of unknown etiology and had a history of bilateral oophorectomy.

Ascending aortic replacement and pulmonary artery patch repair were performed, and the patient was admitted to the ICU under intubation. After surgery, complete paralysis of the right upper and lower limbs was confirmed, and a head CT scan performed on POD 6 revealed cerebral infarction in the left cerebral hemisphere. On POD 7, the patient was extubated. As pericardial fluid accumulation was observed, pericardial fenestration was performed on POD 11, and the patient was re-intubated. Although the patient was re-extubated on POD 13, re-intubation was required on POD 14 owing to her inability to expel sputum. A tracheostomy was performed on POD 16.

**Fig. 3:**
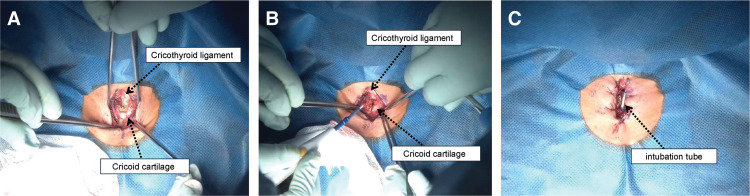
Cricotracheostomy. (**A**) After making a vertical incision, the cricothyroid ligament and cricoid cartilage were exposed. (**B**) After making a midline incision at the cricoid cartilage with an electrocautery knife, its anterior portion was removed. (**C**) A fistula was created with 8 stitches around. The cuff of the intubation tube was shifted proximally to prevent damage during the procedure and to prevent bleeding around the fistula. The intubation tube was removed after completing fistula creation and replaced with a tracheostomy tube.

Cricotracheostomy was selected because the patient had a low-lying larynx, and a conventional tracheotomy would have caused communication with the sternal wound, raising serious concerns about wound infection. Cricotracheostomy was performed as follows: (1) a 40-mm vertical skin incision was made in the midline of the cricoid cartilage, (2) the anterior portion of the cricoid cartilage was exposed by detaching the cricothyroid muscle, (3) the cricoid cartilage was carefully separated from the underlying perichondrium, (4) the anterior portion of the cricoid cartilage was removed, (5) the intubation tube was pushed proximally about 20–30 mm before making the incision in the perichondrium, (6) a horizontal H-shaped incision was made with a scalpel from the cricothyroid ligament to the perichondrium under cricoid cartilage, (7) 8 stitches were placed around the fistula with 3-0 VICRYL (Ethicon, Somerville, NJ, USA), and (8) the tracheostomy tube was inserted after removing the intubation tube (**Fig. 3**).

**Fig. 4:**
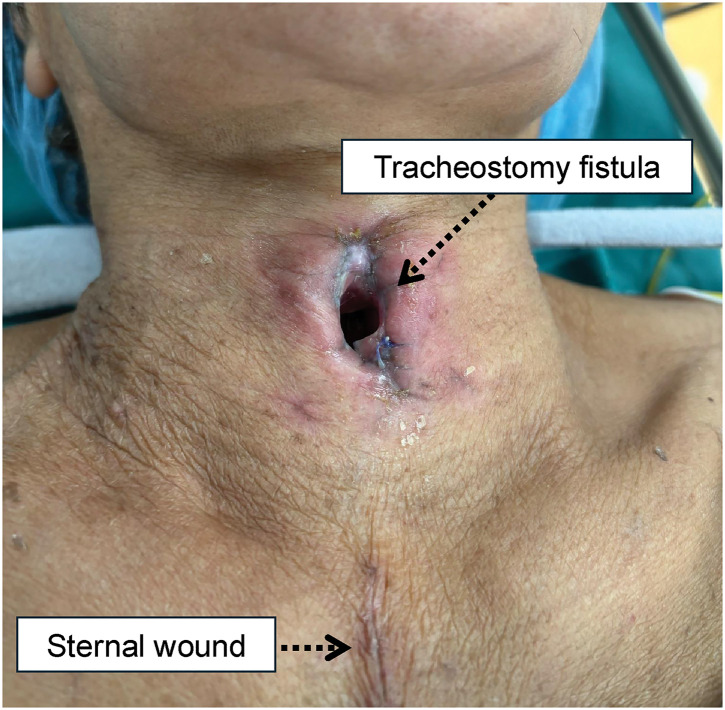
Completed fistula. The fistula was well-spaced from the sternal wound.

Although cricotracheostomy was basically performed following Kano’s original method,^5)^ considering the fragility of the patient’s tissues and hemostasis, the anterior cricoid cartilage was removed using an electric scalpel instead of a bone rongeur forceps Luer. In addition, we devised a method to push the intubation tube proximally before creating a fistula to prevent damage to the cuff and for the aspiration of blood during the procedure. Furthermore, we used absorbable sutures around the fistula in case the stitches were difficult to remove. No postoperative complications were observed. On POD 30, the first tracheal cannula exchange was performed without complications. The completed fistula was well-spaced from the sternal wound (**Fig. 4**).

**Fig. 5:**
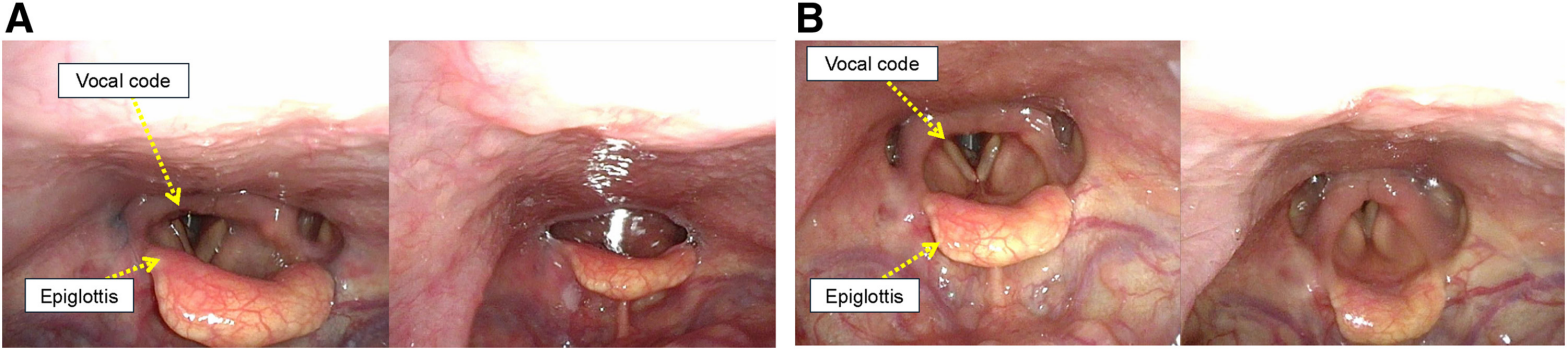
Videoendoscopic evaluation of swallowing. (**A**) The epiglottis functioned normally, and no significant aspiration was observed. (**B**) There were no problems with vocal cord movement.

**Fig. 6:**
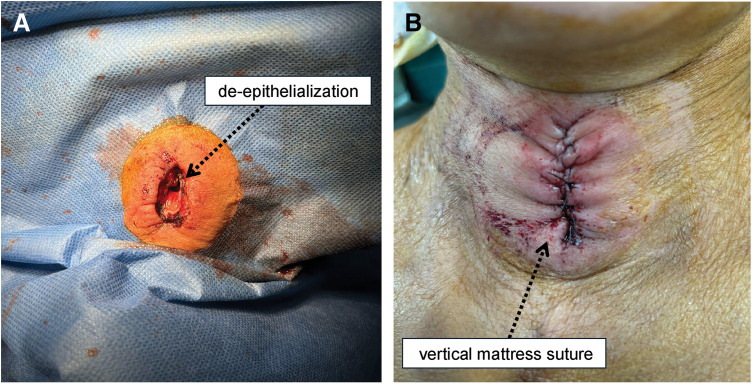
Closure of the fistula. (**A**) De-epithelialization was performed around the fistula. (**B**) Fistula after closure.

**Fig. 7:**
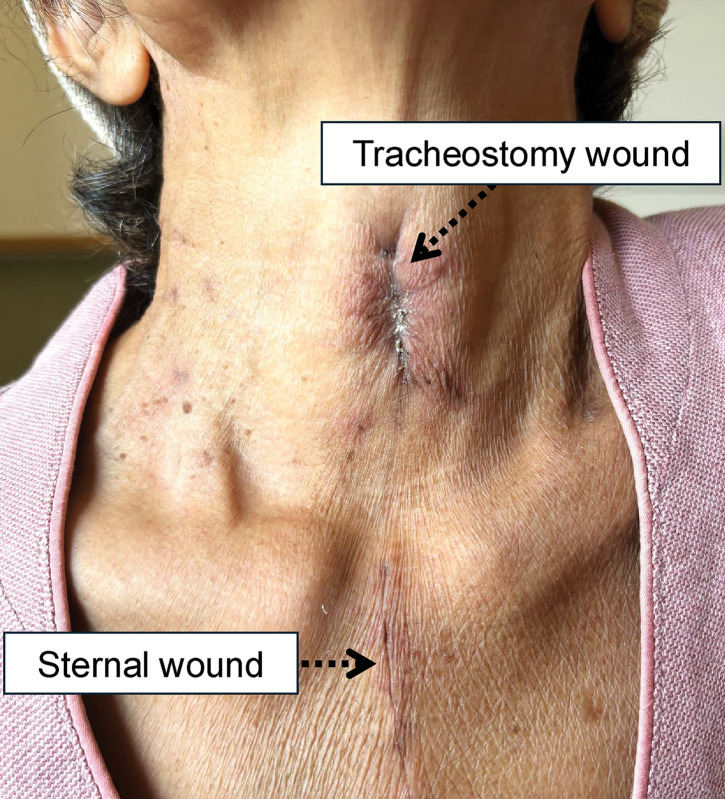
Tracheal fistula after suture removal and sternal wound. The wound was clean and completely free of infection.

**Fig. 8:**
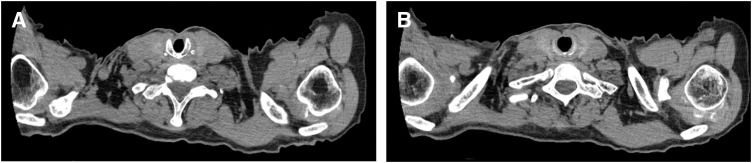
CT performed 120 days after cricotracheostomy. (**A**) The anterior portion of the cricoid cartilage was removed, and the absence of granulation formation and stenosis was confirmed at the removed portion. (**B**) The absence of tracheal stenosis was also confirmed below the 1st tracheal cartilage.

A videoendoscopic evaluation of swallowing was performed on POD 39. Swallowing and vocal cord functions were almost normal (**Fig. 5**). Weaning progressed gradually, and the patient was completely weaned off mechanical ventilation on POD 41. Primary closure of the cricotracheostomy fistula was performed on POD 47 using the Kobari method.^9)^ After de-epithelialization around the fistula, closure was performed using a vertical mattress suture of 3-0 Nylon (**Fig. 6**). Immediately after fistula closure, the patient had no speech or swallowing problems. The patient remained ambulatory with residual right hemiplegia and was transferred to another hospital for rehabilitation on POD 72. No complications associated with the cricotracheostomy or fistula closure were observed (**Fig. 7**). After discharge from another hospital on POD 119, the patient visited a clinic for follow-up on POD 136, and no subglottic stenosis was confirmed on CT (**Fig. 8**).

## DISCUSSION

Tracheotomy after cardiac surgery requires considerations different from those in the general population. The incidence of SWI in patients who underwent tracheostomy after cardiac surgery was high (7%), and infection and short-term mortality rates were not significantly different between the ST and PDT groups.^4)^ Moreover, tracheostomy was an independent risk factor for post-sternotomy deep SWI, which is a predictor of mortality.^10)^

Regarding the timing of tracheotomy and its impact on SWI, one systematic review reported no significant difference in the incidence of SWI between early (<POD 14) and late (≥POD 14) tracheostomy in ST and PDT ^4)^. The incidence of SWI in very early tracheotomy (≤POD 7) varies. Two studies showed that no SWIs occurred in 15^3)^ and 57^11)^ patients who underwent very early ST, and the average timing of performing ST was POD 4.8 (standard deviation, 0.9) and POD 5.6 (standard deviation, 0.7), respectively. In contrast, one study showed that the frequency of SWI in very early PDT was 16% (14 of 109 patients), with no significant difference from prolonged intubation ^12)^. Overall, there are few comparative studies evaluating the incidence of SWI between ST and PDT in the context of very early tracheotomy.

Traditionally, ST has been performed at the level of the 1st to 2nd (or 3rd) tracheal cartilage, and ST involving the cricoid cartilage is considered contraindicative for its potential complications, such as subglottic stenosis and difficulty in cannula removal^13,14)^. Cricotracheostomy is a novel method of ST, mainly performed by otolaryngologists in cases of anatomical abnormalities such as a low-lying larynx, obesity, short neck, aberrant course of the brachiocephalic artery, and thyroid tumors.^5,15–17)^ The removal of the cannula is not very difficult.^16)^ Although there are a few reports on the rate of subglottic stenosis after cricotracheostomy, subglottic stenosis after tracheostomy mostly follows granulation tissue formation.^18)^ A previous study reported that the percentage of granulation tissue formation after cricotracheostomy was 20.0%.^19)^ This percentage is consistent with previously reported rates of granulation tissue formation after tracheostomy (1.5%–26%).^20–23)^ It is also known that subglottic stenosis in conventional tracheostomy rarely causes clinical problems^24)^; thus, the rate of this complication may be overestimated.^25)^ Moreover, only a few clinically significant cases of subglottic stenosis after cricotracheostomy have been reported. If subglottic stenosis does occur, it usually develops within 3–6 weeks after tracheostomy.^26)^ Our patient had no symptomatic subglottic stenosis until 90 days after cricotracheostomy. Therefore, based on available literature on conventional tracheostomy, the probability of future occurrence of subglottic stenosis is not high. However, the cricothyroid muscle attached to the side of the cricoid cartilage stretches the vocal cords and is essential for high-pitched phonation. Cricotracheostomy involves the detachment of the cricothyroid muscle from the cricoid cartilage, raising concern that high-pitched phonation may be hindered after surgery.^8)^ We believe that for patients who have undergone cardiac surgery, cricotracheostomy is more effective than conventional ST or PDT in reducing the risk of SWI for the following reasons. First, the tracheostomy fistula can be created at a location distant from the sternal wound. With cricotracheostomy, a fistula can be securely separated from the sternal wound without requiring any manipulation of the thyroid gland^5)^ or its caudal side, such as dissection, and therefore there is minimal concern about communication with the sternal wound. Second, there are advantages related to the diameter and stability of the fistula. The fistula created with cricotracheostomy is supported on both sides by the cricoid cartilage and sutured circumferentially, making it extremely stable. By adjusting the extent of cricoid cartilage resection depending on the outer diameter of the cannula to be inserted, the gap between the cannula and fistula can be reduced. This decreases sputum contamination outside the fistula, which inevitably reduces contamination of the sternal wound. Furthermore, we believe that cricotracheostomy has advantages over conventional ST and PDT and can be performed even on POD 2–3. As the disinfection area during cricotracheostomy is located at a distance from the sternal wound, it may be possible, depending on the situation, to perform the procedure without removing the sternal wound dressing. This means that contamination of the sternal wound during surgery can be minimized, and cricotracheostomy may allow tracheostomy to be performed with a reduced risk of SWI compared with that in conventional ST or PDT, even immediately after cardiac surgery. Therefore, cricotracheostomy may shorten the time to tracheostomy after cardiac surgery. Although there are some reports of this procedure being performed in patients who have undergone cardiac surgery,^8)^ reports of its detailed outcomes are scant. Moreover, few studies have examined the incidence of SWI after cricotracheostomy and cardiac surgery, and even case reports have not reported detailed outcomes. Therefore, the above hypothesis requires further verification in future studies.

In our case, anatomical abnormalities, such as a low-lying larynx, were also found; however, cricotracheostomy was selected mainly considering the possibility of SWI. The conventional ST site was considered too close to the sternal wound, and communication was a serious concern. The created tracheal fistula was located at a sufficient distance from the sternal wound, and this prevented communication with the wound and avoided SWI. As cricotracheostomy fistulas are less likely to close spontaneously after cannula removal compared with those of conventional STs, a closure procedure was performed. It had no effect on swallowing, and speech function was completely maintained. A limitation of our case report is that it presents the findings of a single case with a favorable outcome, and further case studies are needed for generalization. Although additional case accumulation is necessary, the method described is worth considering for use in patients after cardiac surgery at risk of SWI.

## CONCLUSIONS

We present a case of successful primary closure of a cricotracheostomy fistula after cardiac surgery. Our patient had a low-lying larynx, and SWI caused by the tracheostomy was a serious concern. No SWI was observed, and speech function was completely maintained after fistula closure. Cricotracheostomy may be a good option for patients after cardiac surgery. To confirm the usefulness of this procedure, future comparative studies of SWI incidence between conventional ST and PDT will be necessary in patients undergoing tracheostomy after cardiac surgery.

## References

[1] Ben-Avi R, Ben-Nun A, Levin S, et al. Tracheostomy after cardiac surgery: timing of tracheostomy as a risk factor for mortality. J Cardiothorac Vasc Anesth 2014; 28: 493–6. 24525162 10.1053/j.jvca.2013.10.031

[2] Robertson DS. Tracheostomy and open heart surgery. Proc R Soc Med 1964; 57: 855–64. 14208049 10.1177/003591576405700943 PMC1898731

[3] Okada M, Watanuki H, Masato T, et al. Impact of tracheostomy timing on outcomes after cardiovascular surgery. J Cardiothorac Vasc Anesth 2022; 36(8 Pt A): 2335–8. 34756803 10.1053/j.jvca.2021.10.001

[4] Toeg H, French D, Gilbert S, et al. Incidence of sternal wound infection after tracheostomy in patients undergoing cardiac surgery: a systematic review and meta-analysis. J Thorac Cardiovasc Surg 2017; 153: 1394–1400.e7. 27964980 10.1016/j.jtcvs.2016.11.040

[5] Kano M, Hasegawa H. Cricotracheostomy: new technique to open an airway in emergencies with partial resection of the cricoid cartilage. Br J Oral Maxillofac Surg 2017; 55: 84–5. 27236208 10.1016/j.bjoms.2016.05.004

[6] Toyosaki M, Nakama T, Tamashiro M, et al. Cricotracheostomy in a patient with severe spinal cord injury: its usefulness in trauma patients requiring critical care. Cureus 2025; 17: e89993. 40951107 10.7759/cureus.89993 PMC12428930

[7] Naito K, Chida I, Akizuki H. Two cases undergoing tracheostomaplasty by resection of the cricoid cartilage (in Japanese with English abstract). Tokushima Red Cross Hosp Med J 2017; 22: 99–103.

[8] Nakasone W, Maeda H, Suzuki M. Clinical review of patients undergoing cricotracheostomy (in Japanese with English abstract). Jibi inkoka Tenbou 2023; 66: 24–8.

[9] Kobari T, Kano M, Sato H, et al. Successful primary closure of the crico-tracheostomy stoma: a report of four cases (in Japanese with English abstract). J Jpn Soc Head Neck Surg 2016; 26: 227–33.

[10] Tsai YC, Phan K, Stroebel A, et al. Association between post-sternotomy tracheostomy and deep sternal wound infection: a retrospective analysis. J Thorac Dis 2016; 8: 3294–300. 28066609 10.21037/jtd.2016.11.70 PMC5179377

[11] Gaudino M, Losasso G, Anselmi A, et al. Is early tracheostomy a risk factor for mediastinitis after median sternotomy? J Card Surg 2009; 24: 632–6. 20078708 10.1111/j.1540-8191.2009.00907.x

[12] Trouillet JL, Luyt CE, Guiguet M, et al. Early percutaneous tracheotomy versus prolonged intubation of mechanically ventilated patients after cardiac surgery: a randomized trial. Ann Intern Med 2011; 154: 373–83. 21403073 10.7326/0003-4819-154-6-201103150-00002

[13] Bennett JDC. High tracheostomy and other errors—revisited. J Laryngol Otol 1996; 110: 1003–7. 8944871 10.1017/s0022215100135625

[14] Tavin E, Singer L, Bassila M. Problems in postoperative management after anterior cricoid split. Arch Otolaryngol Head Neck Surg 1994; 120: 823–6. 8049042 10.1001/archotol.1994.01880320025006

[15] Kasahara K, Nishiyama T, Shigetomi S, et al. Cricotracheostomy in a patient with severe kyphosis: a case report. Ear Nose Throat J 2024; 103: NP633–6. 35188407 10.1177/01455613221077596

[16] Murono S, Kakinouchi K, Imaizumi M, et al. Decannulation after cricotracheostomy: a comparison of partial cricoid cartilage resection with conventional tracheostomy. Acta Otolaryngol 2021; 141: 403–7. 33512264 10.1080/00016489.2021.1871645

[17] Kakizaki T, Tsubuku T, Tsushima N, et al. Tracheostomaplasty by partial resection of the cricoid cartilage (in Japanese with English abstract). J Jpn Soc Head Neck Surg 2012; 22: 87–92.

[18] Zias N, Chroneou A, Tabba MK, et al. Post tracheostomy and post intubation tracheal stenosis: report of 31 cases and review of the literature. BMC Pulm Med 2008; 8: 18. 18803874 10.1186/1471-2466-8-18 PMC2556644

[19] Nanjo K, Ueha R, Dealino MA, et al. A retrospective study of cricotracheostomy: indications, techniques, and clinical outcomes. Otolaryngol Head Neck Surg 2025; 172: 953–9. 39523566 10.1002/ohn.1053 PMC11844326

[20] Van Buren NC, Narasimhan ER, Curtis JL, et al. Pediatric tracheostomy: timing of the first tube change. Ann Otol Rhinol Laryngol 2015; 124: 374–7. 25432165 10.1177/0003489414560430

[21] Carron JD, Derkay CS, Strope GL, et al. Pediatric tracheotomies: changing indications and outcomes. Laryngoscope 2000; 110: 1099–104. 10892677 10.1097/00005537-200007000-00006

[22] Carr MM, Poje CP, Kingston L, et al. Complications in pediatric tracheostomies. Laryngoscope 2001; 111: 1925–8. 11801971 10.1097/00005537-200111000-00010

[23] Daou CAZ, Chahine EM, Barazi R. Assessment of tracheostomy tube placement and late change practices in an academic tertiary care center. Int Arch Otorhinolaryngol 2024; 28: e407–14. 38974638 10.1055/s-0043-1776723 PMC11226274

[24] Fernandez-Bussy S, Mahajan B, Folch E, et al. Tracheostomy tube placement: early and late complications. J Bronchology Interv Pulmonol 2015; 22: 357–64. 26348694 10.1097/LBR.0000000000000177

[25] James P, Parmar S, Hussain K, et al. Tracheal stenosis after tracheostomy. Br J Oral Maxillofac Surg 2021; 59: 82–5. 33160732 10.1016/j.bjoms.2020.08.036

[26] Koitschev A, Simon C, Blumenstock G, et al. Suprastomal tracheal stenosis after dilational and surgical tracheostomy in critically ill patients. Anaesthesia 2006; 61: 832–7. 16922748 10.1111/j.1365-2044.2006.04748.x

